# The Medical Society of Virginia

**Published:** 1873-01

**Authors:** 


*The Medical Society of Virginia.*

The third annual session of this Society was held in Staunton, Virginia. The meeting was called to order by the President, Dr. A. M. Fauntleroy, of Staunton. Addresses were delivered by Hon. A. H. H. Stuart, Dr. Samuel Kennerly, and Dr. Landon B. Edwards.

The following interesting papers were read during the session, and were sometimes the subject of warm and earnest discussion:

An essay, by Dr. Payne, of Fauquier, on the "topography, hydrography, petrology and climatology, with a history of the endemic and epidemic diseases of the Piedmont region of Virginia, from the year 1846 to 1872 inclusive." As this was a very long paper, only that portion of it applying to the treatment of typhoid fever was read.

One by Dr. Gray, of Fluvanna, on the hypodermic use of sulphate of strychnia as an optic nerve stimulant.

Dr. Parker, of Richmond, read a very interesting paper on the subject of sewing machines and their effect on the health of females. His conclusions were as follows:

1. That fatigue is not disease, and that there is no reason to conclude that the use of the muscles employed in machine work, for a reasonable time, is injurious.

2. That the machine may be used for four or five hours daily in a family, by a lady in ordinary health, without injury.

3. That the damage to health in the factory is due to the hygienic conditions under which the work is done, and the natural delicacy of some of the operatives, unfitting them for long-continued labor of any kind.

4. That the sewing machine is a great boon to womankind, increasing her compensation, protecting her sight, and, in the family, lessening her labors.

A paper by Dr. Cabell, urging the Legislature to make an appropriation to carry out the objects for which the State Board of Health was created.

A paper by Dr. J. H. Claiborne, on diphtheria.

The following officers were elected for next year: President, Dr. Harvey Black, of Blacksburg; First Vice-President, Dr. A. S. Payne, Fauquier; Second Vice-President, Dr. H. Latham, Lynchburg; Third Vice-President, Dr. R. W. Burgess, Norfolk; Fourth Vice-President, Dr. J. H. Claiborne, Petersburg; Fifth Vice-President, Dr. Samuel Kennerly, Augusta; Sixth Vice-President, Dr. O. Fairfax, Richmond; Recording Secretary, Dr. L. B. Edwards, Richmond; Corresponding Secretary, Dr. Frank D. Cunningham, Richmond; Treasurer, Dr. W. W. Parker, Richmond.

The new standing committees are: Committee on Publication--Drs. A. A. Tebault, Princess Anne; J. L. Cabell, University of Virginia; M. P. Christian, of Lynchburg, and the Recording and Corresponding Secretaries. Executive Committee—Drs. W. W. Parker, Richmond; W. F. Figgart, Christiansburg; S. C. Gleaves, Wytheville; W. D. Hooper, Liberty; G. McDonald, Union, Monroe county; L. B. Edwards, Richmond.

Dr. Stribling was elected an honorary member of the Society.

Norfolk was selected as the place for the next meeting, and the time the second Tuesday in November, 1873.

Dr. R. S. Hamilton was elected Orator for next year.

We are indebted to Dr. F. Horner for papers giving the proceedings of the Society.

